# Interannual changes in zooplankton echo subtropical and high latitude climate effects in the southern East China Sea

**DOI:** 10.1371/journal.pone.0197382

**Published:** 2018-05-31

**Authors:** Juan Carlos Molinero, Li-Chun Tseng, Celeste López Abbate, Eduardo Ramirez-Romero, Jiang-Shiou Hwang

**Affiliations:** 1 GEOMAR—Helmholtz Center for Ocean Research, Kiel, Germany, Marine Ecology/Food Webs, Duesternbrooker Weg 20, Kiel, Germany; 2 Institut de Recherche pour le Développement (IRD), MARBEC, IRD/CNRS/IFREMER/Univ Montpellier, France; 3 Institute of Marine Biology, National Taiwan Ocean University, Keelung, Taiwan; 4 Center of Excellence for the Oceans, National Taiwan Ocean University, Keelung, Taiwan; 5 Instituto Argentino de Oceanografía (CONICET-UNS), Camino La Carrindanga, Bahía Blanca, Argentina; 6 Instituto Mediterráneo de Estudios Avanzados, IMEDEA (CSIC-UIB), Fish Ecology Group, C/Miquel Marqués 21, Esporles, Illes Balears, Spain; Zhejiang University College of Life Sciences, CHINA

## Abstract

Climate variability plays a central role in the dynamics of marine pelagic ecosystems shaping the structure and abundance changes of plankton communities, thereby affecting energy pathways and biogeochemical fluxes in the ocean. Here we have investigated complex interactions driven a climate-hydrology-plankton system in the southern East China Sea over the period 2000 to 2012. In particular, we aimed at quantifying the influence of climate phenomena playing out in tropical (El Nino 3.4) and middle-high latitudes (East Asia Winter Monsoon, EAWM, and Pacific Decadal Oscillation, PDO) on pelagic copepods. We found that the EAWM and El Nino 3.4 showed a non-stationary and non-linear relationship with local temperature variability. In the two cases, the strength of the relationship, as indexed by the wavelet coherence analysis, decreased along with the positive phase of the PDO. Likewise, the influence of EAWM and El Nino3.4 on copepods exhibited a non-stationary link that changed along with the PDO state. Indeed, copepods and EAWM were closely related during the positive phase, while the link copepods–El Nino 3.4 was stronger during the negative phase. Our results pointed out cascading effects from climate to plankton driven by the positive phase of the PDO through its effect on temperature conditions, and likely through a larger southward transport of nutrient-rich water masses to northern Taiwan and the Taiwan Strait. We suggest a chain of mechanisms whereby the PDO shapes interannual dynamics of pelagic copepods and highlight that these results have implications for integrative management measures, as pelagic copepods plays a prominent role in food web dynamics and for harvested fish in the East China Sea.

## Introduction

Climate variability plays a central role in energy pathways and biogeochemical fluxes in the ocean. By sculpting the physical environment and nutrient dynamics, climate shapes the structure and abundance of plankton communities [1]. Such changes affect the entire food web dynamics, and ultimately the abundance of higher trophic levels over interannual scales. Underlying mechanisms driving such temporal trends are however context dependent and vary across spatial and temporal scales [2, 3]. Hence, resolving how climate cascades down affecting the pace of food web dynamics is fundamental to understand and model marine food web dynamics under global change scenarios. This may help forecasting ecological responses to global changes, eventually shading light on the long term sustainable use of coastal resources [4].

The East China Sea (ECS) is one of the most productive marginal seas in the world. This region experiences the influence of hemispheric-wide climate phenomena, i.e. El Niño‐Southern Oscillation (Nino), East Asia Winter Monsoon (EAWM) and Pacific Decadal Oscillation (PDO) that shape regional climate patterns over interannual and decadal scales. Historically, the ECS has been one of the world’s major fishing grounds, mainly fostered by the influence of both Yangtze River discharges and long-shore currents, the China Coastal Current and the Kuroshio Current [5]. The high nutrient load associated with the above hydrographic features boosts large primary and secondary production and favor environmental conditions for fish larvae [6]. Projected scenarios of global change, however, forewarn on changes in climate (i.e. warming, wind patterns) with potential implications for East Asia marine and terrestrial ecosystems [7].

We here examine large scale climate influence on the physical environment and plankton communities in the ECS. We aimed at quantifying the influence of tropical (El Nino 3.4) and middle-high latitudes (East Asia Winter Monsoon, EAWM, and Pacific Decadal Oscillation, PDO) climate phenomena on pelagic copepods in the southern East China Sea over the period 2000 to 2012. Pelagic copepods contribute to a large proportion of the zooplankton biomass, they are prominent component of food webs in the continental shelf and directly affect both biogeochemical cycling and fish larvae survival. Hence, marked interannual variations in their abundance are likely to affect not only higher trophic levels, but the pace of carbon cycling as well.

## Data and methods

### Physical and biological data

**Fig 1:**
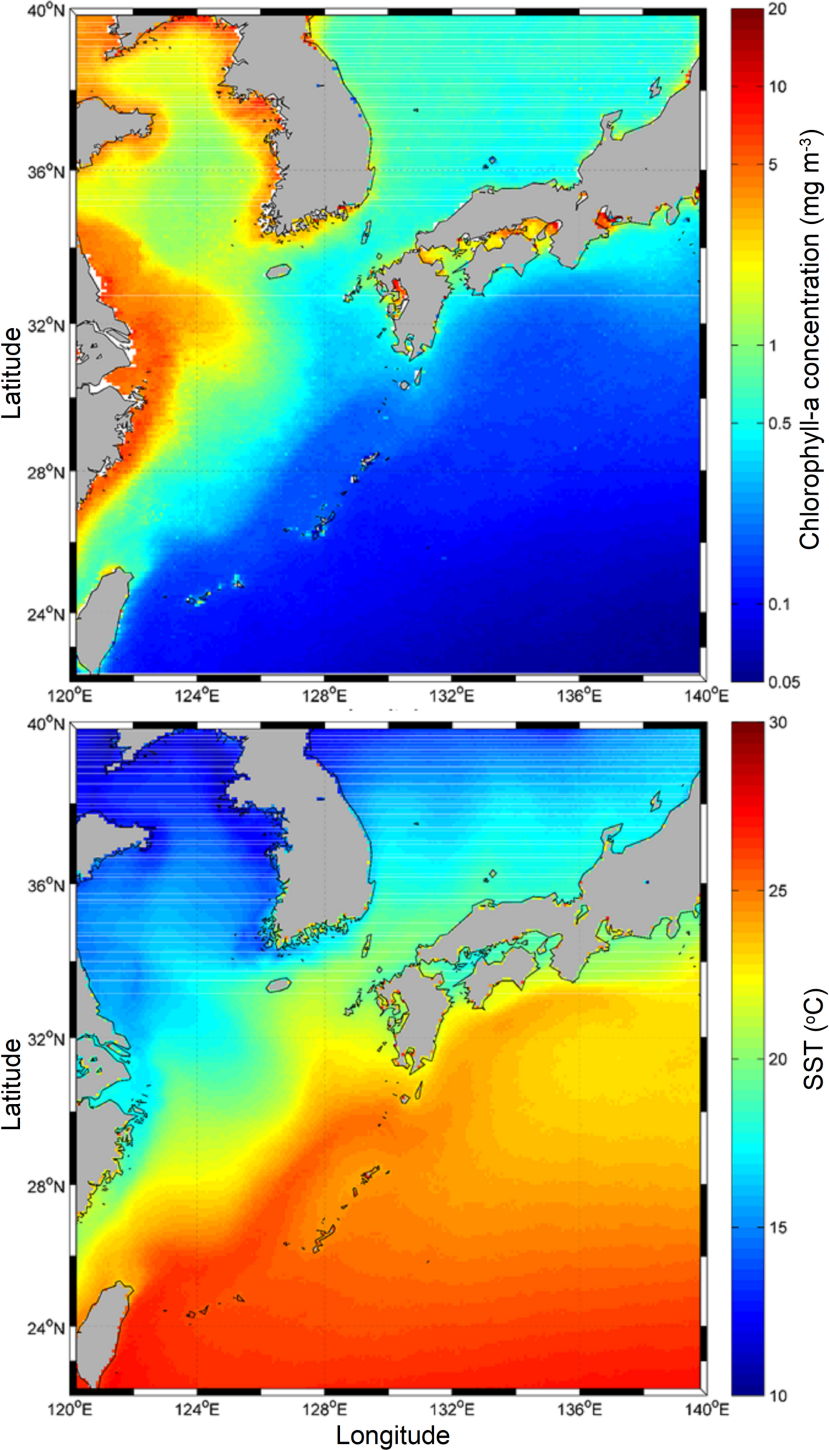
Regional conditions of sea surface temperature (SST) and surface chlorophyll concentration in the East China Sea. Satellite images of sea-surface temperature and surface chlorophyll in the East China Sea. Colour scale in each panel display SST in °C and chlorophyll in mg.m^-3^. Sampling locations are indicated in the map.

The area investigated is located in the southern margin of the ECS, northern Taiwan coast (Fig 1). Standardized modes of low-frequency atmospheric variability having an impact in the West Pacific, e.g. El Nino, EAWM and PDO, were used to assess the climate signal on the East China Sea. These climate proxies shape atmospheric conditions in the north and subtropical western Pacific Ocean [8]. Regional sea surface temperature (SST) in the southern ECS, area extending from 29° to 23°N and from 121° to 129°E (Fig 1), was evaluated by using monthly anomalies with regards to the period 1950–2012 from the Climate Diagnostics Center (NCEP/NCAR) reanalysis fields [9].

Collection of biological data was done seasonally onboard the Ocean Research Vessel II National Taiwan Ocean University. Each cruise sampled 26 stations covering an area extending ca. 70 km2 (25.3 to 25.05°N and from 121.2 to 121.5°E). Depth of sampling stations was on average 42 m (minimum depth 16 m, and maximum depth 80 m). A total number of 1621 samples were collected from 1998 to 2012. Chlorophyll fluorescence was obtained prior the zooplankton collection in the upper 25 m depth, or in the entire water column in shallower stations, using a CI Fluorometer (SBE sensor) fenced to the Sea-Bird CTD multiparameter sonde. Zooplankton samples were collected by surface net tows in the upper 0–5 m depth layer using a zooplankton net (180 cm long, 0.45 m mouth diameter, 333 μm mesh) with a Hydrobios flowmeter towed for 10 min at a constant speed of 2 knots. Samples were preserved in seawater with 5% buffered formaldehyde immediately after collection. We acknowledge that the surface sampling does not provide accurate estimates of zooplankton abundances, however the consistency of the sample treatment throughout the whole period makes samples comparable among them and the temporal patterns of these data, therefore, might be indicative of changes in the zooplankton community. Abundances were averaged by cruise to create the plankton time series. Missing data due to bad weather conditions were estimated using the best fitting state-space model based on the Kalman filter [10].

### Statistical analysis

Temporal trends were removed from the standardized monthly times-series and residuals were used for analysis. As climate variables are non-stationary we used wavelet analysis (Continuous Wavelet Transform, CWT) to assess the time-varying signal of each climate phenomena, i.e. El Nino, EAWM and PDO. Subsequently, to quantify their correlation with the local sea surface temperature in the time frequency space we used the wavelet coherence method (CWT), which performs a local time-scale decomposition of time series quantifying its spectral characteristics as a function of time [11]. We used the Morlet wavelet function, which best describes time series with unknown frequencies allowing a better separation of the phase and the amplitude of the studied signal [11]. The 5% statistical significance level was determined by using bootstrap simulations (1000 times) considering a first order autoregressive process with lag-1 autocorrelation. The statistical significance was assessed relative to the null hypotheses that the signal is generated by a stationary process, i.e. mean and variance of the time series do not vary with time [12]. Then, from the CWT of each climate phenomenon we assessed the wavelet coherence to identify areas with high common power and significant links in the time frequency space between the two phenomena. To graphically display the temporal relationship we used only data within the cone of influence.

The quantitative assessment and the identification of predominant factors shaping the abundance of pelagic copepods were achieved by structural equation modeling [13]. The a priori structure of the path model is based on the hypothesis that large scale climate cascades influencing regional climate weather and local hydrological conditions (i.e. SST), both jointly drive the plankton’s physical environment, thereby shaping the structure and functioning of pelagic copepods. Specific effects of climate and environmental variables and their co-variations on plankton structure were assessed using variance partitioning and further explored through path analysis [13]. The strength of the links and the quantification of overall model were determined by simple and partial multivariate regression and Monte Carlo permutation tests (1000 replicates), while the Bayesian Information Criterion (BIC) and Chi-square values were used to assess the robustness of models [14]. The individual path coefficients (i.e. partial regression coefficients) that indicate the strength of the relation between causal and response variables and the fit of the overall path model were evaluated.

## Results

**Fig 2:**
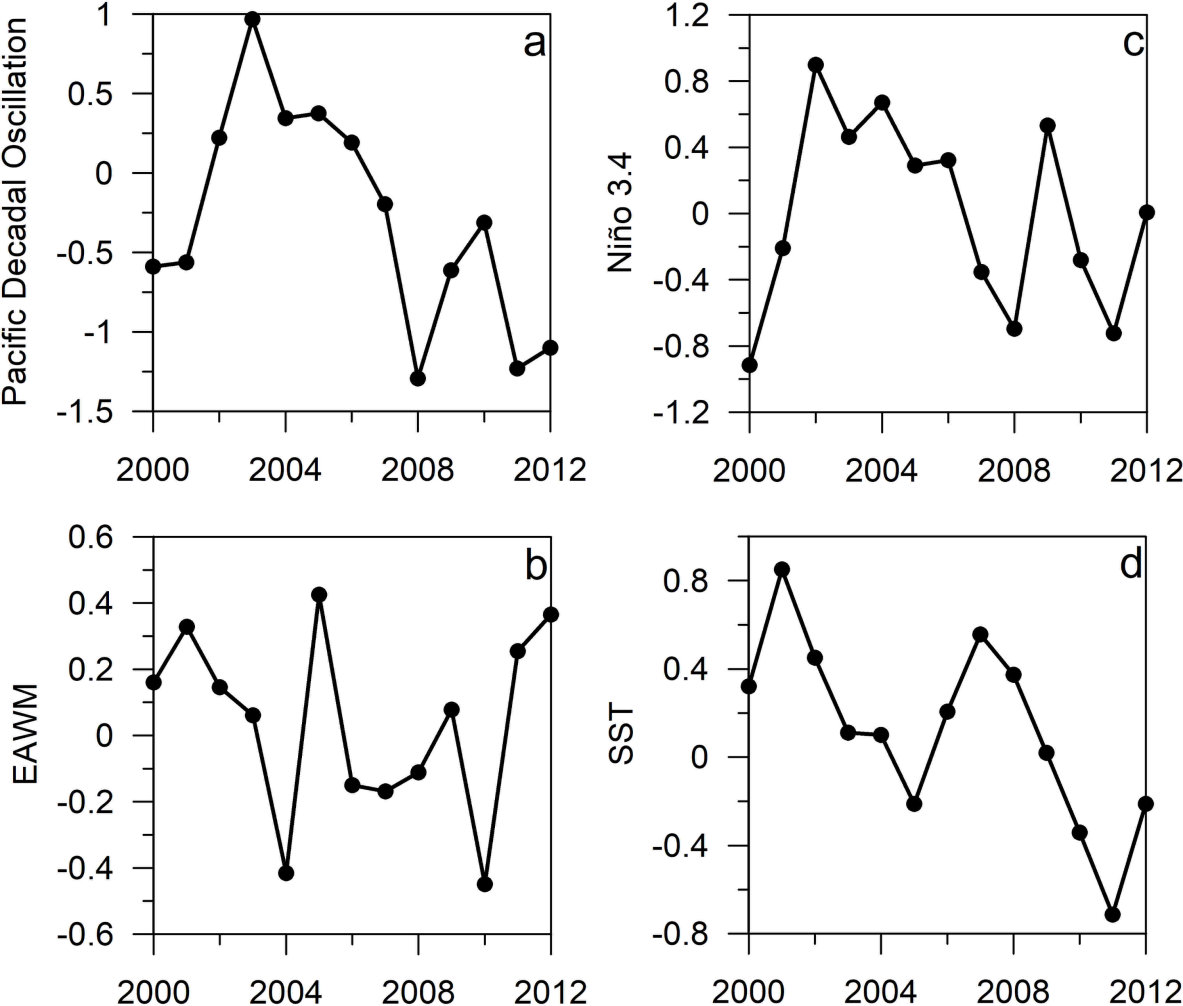
Interannual variability of physical forces shaping the East China Sea marine ecosystem. a) Pacific Decadal Oscillation, b) East Asia Winter Monsoon, c) El Nino3.4, and d) sea surface temperature over the period 2000 to 2012.

**Fig 3:**
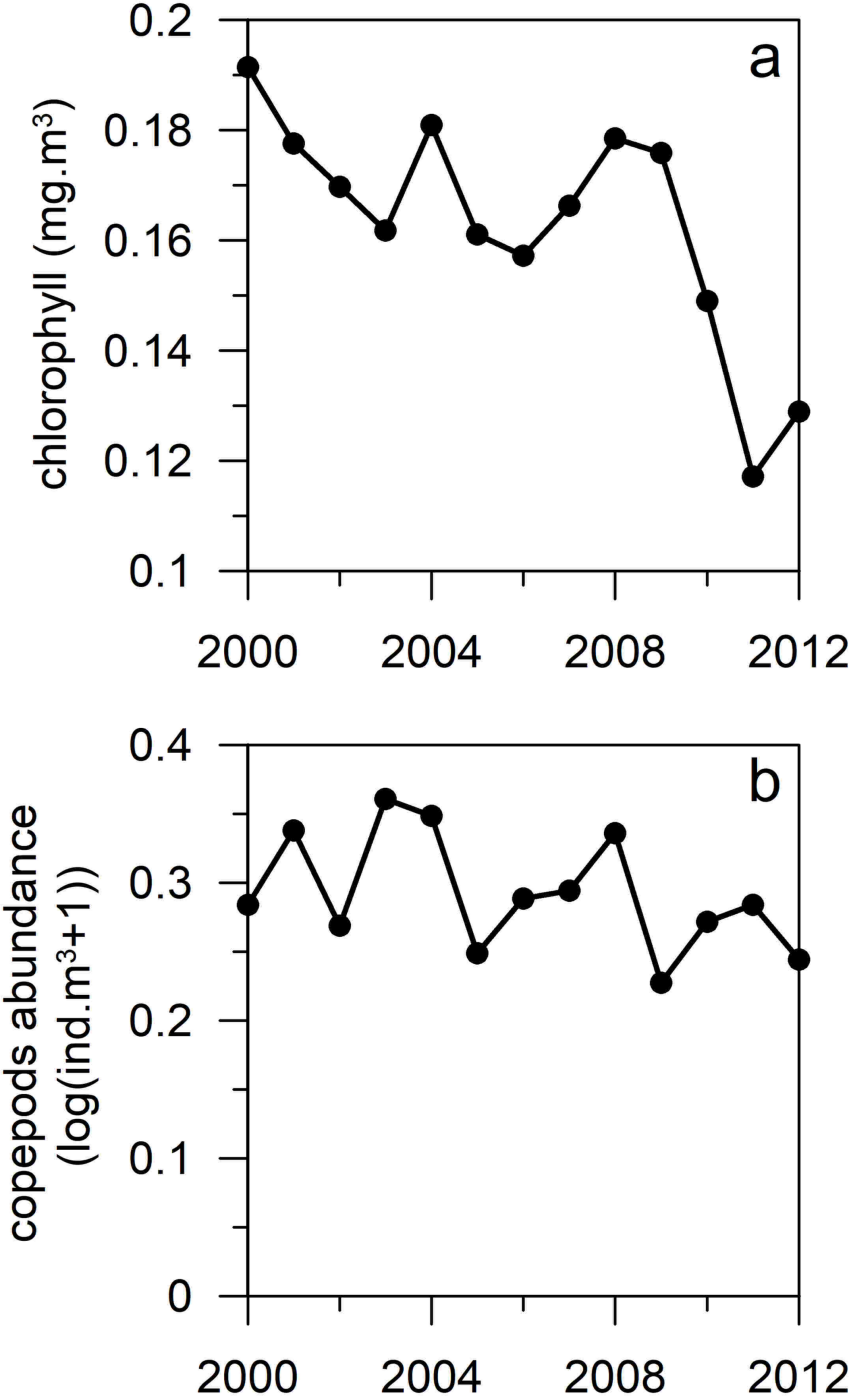
Interannual variability of plankton communities. a) chlorophyll and b) pelagic copepods over the period 2000 to 2012.

Climate phenomena showed marked variations over the period 2000–2012 (Fig 2). Climate signals, PDO and EAWM, governing winter conditions in the East China Sea displayed noticeable changes. The PDO was characterized by a dominance of the positive phase during the years 2002–2007 and shifted afterwards to marked interannual variations mainly dominated by negative values (Fig 2A), whereas the EAWM exhibited large interannual changes in 2004–2005 and 2009-2011(Fig 2B). El Niño 3.4 showed positive values during the early 2000s and in 2009(Fig 2C). In turn, SST in the southern East China Sea showed a general downward pattern with peaks of maxima in 2002 and 2007 (Fig 2D). Likewise, the ecological groups investigated showed a general downward pattern (Fig 3). Chlorophyll concentration displayed higher values in 2000, 2004 and 2008 followed by a marked decline in the years 2010–2012 (Fig 3A), while copepods abundance showed alternate high and low abundance describing a ca. 4-year cycle (Fig 3B).

**Fig 4:**
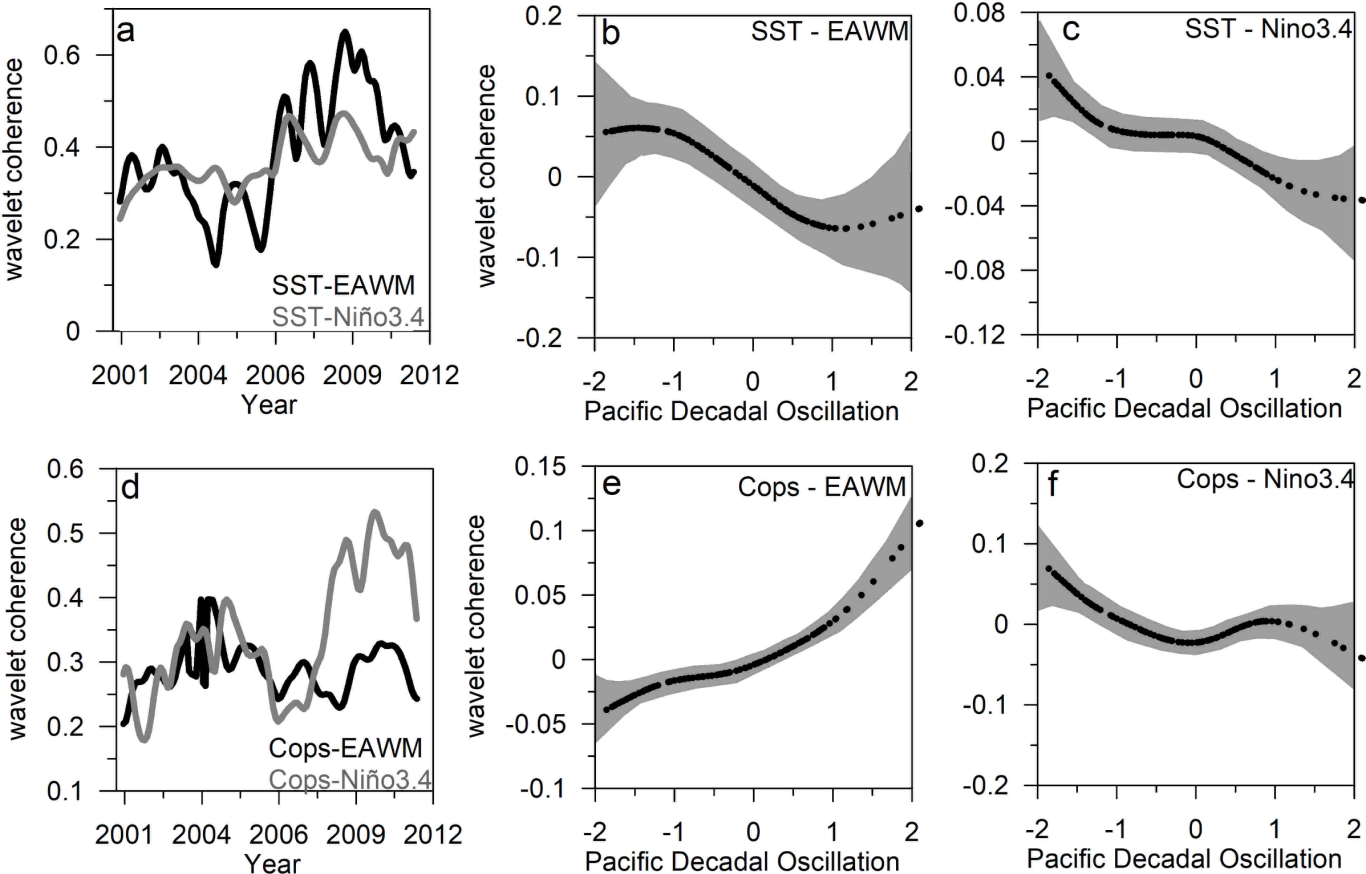
Relationships between regional climate, local temperature and pelagic copepods. a) Wavelet coherence of SST- EAWM and SST-El Nino3.4 in the southern East China Sea. (b and c) Scatter plot of SST-EAWM and SST-Nino3.4 coherence versus the PDO state. (d) Wavelet coherence of copepods-EAWM and copepods-El Nino3.4. (e and f) Scatter plot of copepods-EAWM and copepods-Nino3.4 coherence versus the PDO state.

The assessment of links between climate phenomena, EAWM and El Nino 3.4, and local SST showed a non-stationary relationship that displayed lower coherence over the period 2002–2006 followed by a sharp increase in the two cases, although the change was more prominent in the link EAWM-SST (Fig 4A). These results were projected versus the PDO to identify whether the link between El Nino 3.4 and EAWM with SST change along with the PDO state. GAM models showed a non-linear relationship where the strength of the link, as indexed by the wavelet coherence, decreased in the two cases along with the positive phase of the PDO (Fig 4B and 4C). Similarly, the relationship between copepods and climate (EAWM and El Nino3.4) exhibited a non-stationary link that change along with the PDO state, that is, the higher coherence between copepods and EAWM was observed during the positive phase, while in the link copepods–El Nino 3.4 displayed higher values during the negative phase of the PDO (Fig 4D). As for the link between SST and climate, these relationships showed a non-linear behavior with a sharp increase during the positive phase of the PDO for the link copepods-EAWM. In contrast, higher values were observed during the negative PDO for the copepods-El Nino 3.4 link (Fig 4E and 4F).

**Fig 5:**
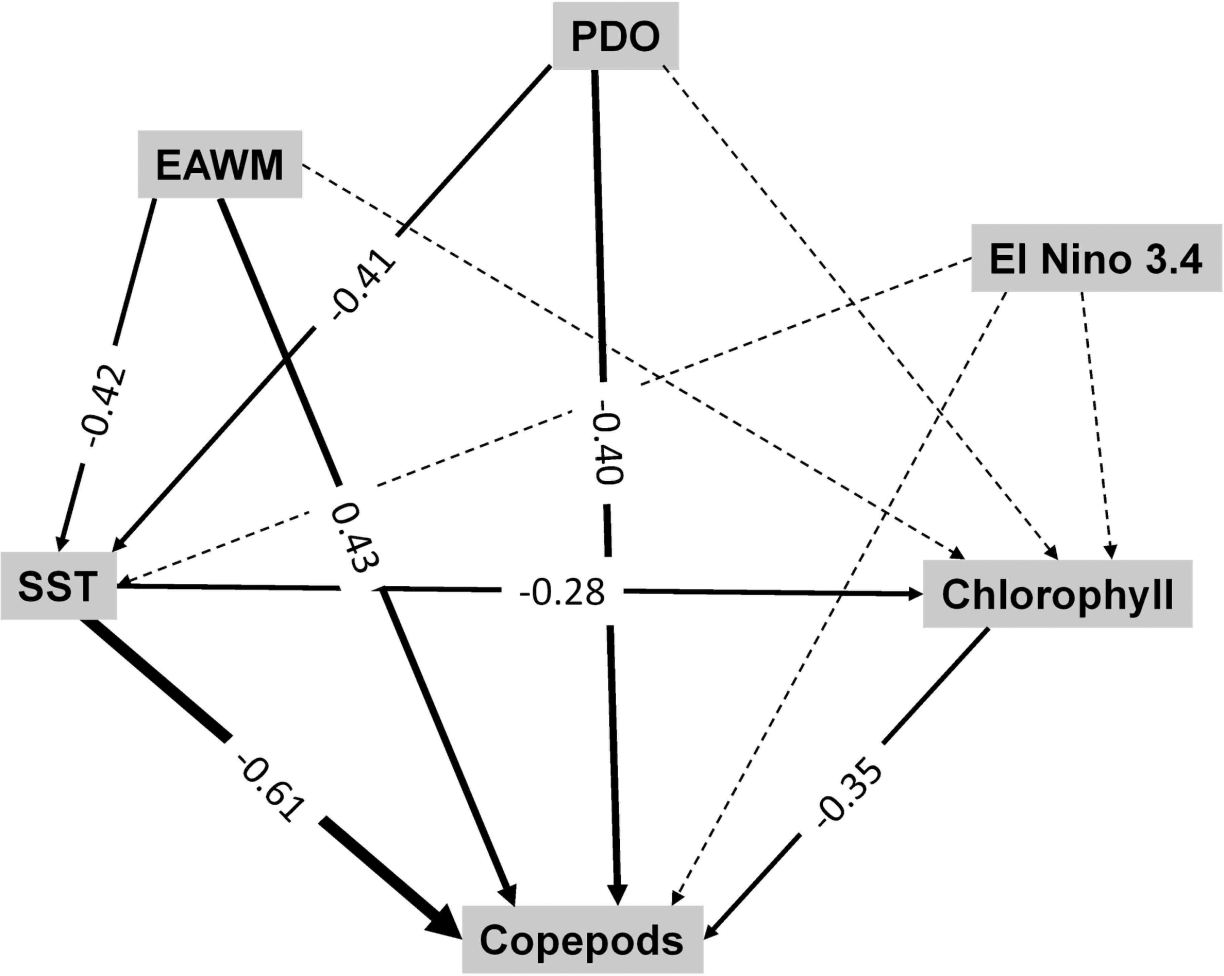
Structural equation model deciphering interactions between climate and pelagic copepods in the southern East China Sea. Path diagram showing cascading effects from climate to pelagic copepods during the positive phase of the PDO in the southern East China Sea over the period 2000 to 2012. Solid paths are statistically significant (P < 0.05) whereas the dashed lines are not. At each significant path the standardized coefficients are represented. The variance explained by the model is 62%, chi-square = 10.84; p<0.01.

Results of the structural equation model showed the main pathways linking climate and plankton, thereby revealing drivers of pelagic copepods in the southern East China Sea (Fig 5). Climate phenomena influence SST in the southern East China Sea (path coefficients: -0.42 and -0.41, respectively for EAWM and PDO). In turn, SST negatively affects copepods abundance and chlorophyll (path coefficients: -0.61 and -0.28, respectively). Copepods abundance were further influenced by climate (path coefficients: 0.43 and -0.40, EAWM and PDO respectively) and by chlorophyll (path coefficient: -0.35).

## Discussion

### Climate influence on environmental conditions of the southern East China Sea

Over the period 2000–2012, the positive phase of the PDO appears as the dominant climate forcing on plankton interannual changes. The atmospheric forcing promoted by the PDO in this region is mainly driven by the enhanced influence of the Aleutian Low dynamics and westerlies during the positive phase of PDO, which favors lower temperatures in the northern and mid-latitude northwestern Pacific, while enhancing SST in the eastern Pacific coast [15]. The PDO has been identified as a major driver of the leading pattern of annual mean SST in the North Pacific. Such influence is particularly noticeable over decadal scales, as the temporal pattern of this climate oscillation displays multi-year periods of stable sign (positive or negative) separated by abrupt sign reversals [16]. However, as shown by our results, the PDO forcing is also noticeable at interannual scales, particularly during winter, when the influence extent to lower latitudes of the Pacific Ocean, such as the southern East China Sea.

The influence of el Nino3.4 and EAWM in this area displayed a non-linear behavior and emphasized a time-varying link that echoes the state of the PDO. For instance, the correlation between SST and EAWM, as indexed by the wavelet coherence analysis, decreased during the positive phase of the PDO and it rose when PDO shifted towards negative values. Similarly, the link between SST and El Nino3.4 slightly increased along with negative PDO values. During the positive phase of the EAWM, the Aleutian Low increase in winter and spring and SST in the East China Sea were closely linked with the PDO state, though the strength of the link is discontinuous. Likewise, we noticed that the governing SST in the equatorial Pacific region, as indexed by the Nino 3.4, varied along with the PDO state and displayed a non-stationary influence on this region.

SST in the southern East China Sea showed marked interannual variations throughout the period 2000 to 2012. These changes are related to the influence of water masses in the northern Taiwan, which closely related to monsoon dynamics and PDO during winter, as shown by our results, although strong El Nino 3.4 events have shown a noticeable impact in the temperature conditions [17]. For instance, El Nino event 1997–1998 fostered anomalously warmer conditions in the region that lasted until 2000 [18] affecting the water column structure and ultimately plankton dynamics. Enhanced stratification decreases nutrient supply and shift nutrient ratios, which determine the dynamics at the base of the plankton food web [19]. Our results highlight the sensitivity of the region to climate phenomena playing out in higher latitudes, i.e. EAWM and PDO. Such climate phenomena shape a large amount of the interannual variance of the water column temperature in the southern East China Sea, as the positive phase of both PDO and EAWM foster the influence of the Aleutian Low dynamics and westerlies, thereby favoring lower temperatures in the northern and mid-latitude northwestern Pacific [20]. The cascading effects from climate to plankton were depicted by SEM results where the strength of the relationships within the climate-plankton network is closely related with the magnitude of climate forcing ascribed to the PDO. This is in agreement with former studies showing that these climate indices and SST further influence the abundance and migration behavior of harvested fish, such as the grey mullet during winter in the Taiwan Strait [21].

These results revealed that such climate influence is mainly ascribed to the effect these atmospheric phenomena have on water temperature and promoting the transport of northern cold waters into the Taiwan Strait. The southward water mass transport mainly occurs in winter, when the intensity of the China Coastal Current increase as consequence of the larger influence of EAWM [22], which is enhanced during the positive phase of the PDO. The increase in the China Coastal Current might foster southward transport from the more productive Yellow Sea and the Bohai Sea [22, 23] to the Taiwan Strait and southern regions thereby promoting favorable conditions for plankton growth. Also, it is worth noticing that the main population centres of some large copepod species, i.e. *Calanus*, are located in northern regions for which enhanced southern transport also contribute with the abundance increase recorded in the southern East China Sea. Likewise, climate-driven water mass dynamics shape the structure and spatial distribution of other relevant zooplankton taxa, such as euphausiids [24] and prominent copepod species, *Calanus sinicus* [18, 24].

### Interannual changes in plankton dynamics under varying climate

We have assessed the climate influence on temporal patterns of pelagic copepods in the southern ECS. Our results provide support for conspicuous climate influence in the southern ECS manifested on the thermal structure of the water column. The climate forcing shapes interannual variations in primary producers, as shown by the close link between SST and chlorophyll. These results are in agreement with former investigations in the northweatern Pacific where evidence has been provided on the climate influence on plankton communities promoting significant changes in phenology and community structure of phytoplankton [25], with earlier blooms during the negative PDO phase, and the abundance of diatoms correlated with thermal conditions from March to April [26]. Similar climate-driven changes in phytoplankton structure have been reported in other oceans. For instance, in the North Atlantic Sea surface temperatures and wind drive phytoplankton structure [27]. Phytoplankton changes bear wide implications in food web trophic dynamics, as they underlie variations in food type for higher trophic levels, and therefore warn that prolonged warming and changing regimes of wind forcing may affect food quality through changes in autotroph communities.

Climate influence shape pelagic communities and appeared manifested in the interannual abundance of pelagic copepods. These results highlight the role high latitude and subtropical climate phenomena shaping the temporal pattern variability of zooplankton in the southern East China Sea. Such close covariation between pelagic copepods and plankton warns on potential changes under scenarios of global warming, as modelling studies forecast potential changes in wind stress and temperature in the North Pacific [28], which might foster northward heat transport from tropical areas to northern Taiwan. Under this environmental scenario copepod southward transport from nutrient-rich cold waters into the Taiwan area might be impaired. Likewise, the major climate driver, the PDO, is forecasted to shift to a higher frequency under global warmer scenarios, where the length of stable periods of similar sing is significantly shortened [29]. Hence, as the positive phase of the PDO is the major driver of copepods interannual changes, such sign variations in the PDO might have noticeable effects on the environmental conditions in the ECS and ultimately on copepod abundance. Gaining knowledge on copepod responses to changing environmental conditions is essential to improve models on food webs dynamics and biogeochemical cycling. These results have implications for integrative management measures, as pelagic copepods plays a prominent role in food web dynamics and for harvested fish.

## Supporting information

S1 File Table A. Interannual variability of climate phenomena shaping the East China Sea marine ecosystem and plankton compartments, chlorophyll, as indicator of phytoplankton, and pelagic copepods. Pacific Decadal Oscillation (PDO), East Asian Winter Monsoon (EAWM), Niño 3.4, Sea Surface Temperature (SST). Table B. Relationships between regional climate, local temperature and pelagic copepods.
(DOCX)
