# Supplementary material for: Interannual changes in zooplankton echo subtropical and high latitude climate effects in the southern East China Sea
Source: PLoS One. 2018 May 31;13(5):e0197382. doi: 10.1371/journal.pone.0197382 (PMC5979015; doi:10.1371/journal.pone.0197382)
Supplement: S1 File — Table A. Interannual variability of climate phenomena shaping the East China Sea marine ecosystem and plankton compartments, chlorophyll, as indicator of phytoplankton, and pelagic copepods. Pacific Decadal Oscillation (PDO), East Asian Winter Monsoon (EAWM), Niño 3.4, Sea Surface Temperature (SST). Table B. Relationships between regional climate, local temperature and pelagic copepods. (DOCX) [file pone.0197382.s001.docx]

**S1 Table A. Interannual variability of climate phenomena shaping the East China Sea marine ecosystem and plankton compartments, chlorophyll, as indicator of phytoplankton, and pelagic copepods. Pacific Decadal Oscillation (PDO), East Asian Winter Monsoon (EAWM), Niño 3.4, Sea Surface Temperature (SST).**

| year | PDO | EAWM | Nino3.4 | SST | Chla | Copepods |
| --- | --- | --- | --- | --- | --- | --- |
| 2000 | -0.43063217 | 0.42318397 | -1.58037945 | 0.4894472 | 1.36840051 | 3.0737554 |
| 2001 | -0.39089433 | 1.01832614 | -0.36223164 | 1.76379025 | 0.70615389 | 0.15688594 |
| 2002 | 0.7410319 | 0.3723594 | 1.5485202 | 0.80318704 | 0.33178574 | -0.47097101 |
| 2003 | 1.82238271 | 0.07397854 | 0.79848919 | -0.01208846 | -0.04560899 | 0.36490913 |
| 2004 | 0.92045425 | -1.61153616 | 1.15523248 | -0.0364331 | 0.86518638 | 0.25289664 |
| 2005 | 0.96380462 | 1.36202508 | 0.49917288 | -0.78673621 | -0.07896487 | -0.65257705 |
| 2006 | 0.69768154 | -0.67125218 | 0.55485963 | 0.21675519 | -0.26225762 | -0.29277364 |
| 2007 | 0.13894348 | -0.73886122 | -0.60760142 | 1.05365481 | 0.16724353 | -0.2427799 |
| 2008 | -1.44575325 | -0.53588685 | -1.20101342 | 0.61759164 | 0.75077121 | 0.13851703 |
| 2009 | -0.46314494 | 0.13266531 | 0.91682355 | -0.23103022 | 0.62528104 | -0.84933271 |
| 2010 | -0.02964128 | -1.73035258 | -0.48404642 | -1.09889575 | -0.65235766 | -0.44777316 |
| 2011 | -1.35664416 | 0.75637053 | -1.24799912 | -1.99058582 | -2.16819602 | -0.33415103 |
| 2012 | -1.16758839 | 1.14898003 | 0.01017354 | -0.78865657 | -1.60743713 | -0.69660563 |

**S2 Table B. Relationships between regional climate, local temperature and pelagic copepods.**

| Year | PDO | SST_EAWM | SST_NINO3.4 | cop_EAWM | cop_NINO3.4 |
| --- | --- | --- | --- | --- | --- |
| 2001 | -0.31 | 0.28185462 | 0.243540012 | 0.203948849 | 0.28100515 |
| 2001 | -0.3 | 0.304555046 | 0.25624719 | 0.207417425 | 0.291921025 |
| 2001 | -0.47 | 0.337664974 | 0.270109663 | 0.221122423 | 0.289770746 |
| 2001 | -1.31 | 0.362573455 | 0.281822557 | 0.241056047 | 0.273524208 |
| 2001 | -0.77 | 0.376974053 | 0.290680437 | 0.257742044 | 0.248352336 |
| 2001 | -1.37 | 0.382196082 | 0.297592961 | 0.266681366 | 0.222375316 |
| 2001 | -1.37 | 0.378219627 | 0.303715206 | 0.269257454 | 0.201659284 |
| 2001 | -1.26 | 0.365322346 | 0.309679286 | 0.269211164 | 0.187838457 |
| 2001 | -0.93 | 0.347028506 | 0.315670105 | 0.269980256 | 0.180103696 |
| 2002 | 0.27 | 0.329071995 | 0.321795516 | 0.273866594 | 0.178392477 |
| 2002 | -0.64 | 0.315569781 | 0.327874597 | 0.281155133 | 0.183668889 |
| 2002 | -0.43 | 0.308514923 | 0.333327171 | 0.288419546 | 0.196845236 |
| 2002 | -0.32 | 0.308454273 | 0.337562004 | 0.290305198 | 0.218006249 |
| 2002 | -0.63 | 0.314111302 | 0.340596611 | 0.286101399 | 0.24440785 |
| 2002 | -0.35 | 0.325150908 | 0.343779593 | 0.281533948 | 0.269587863 |
| 2002 | -0.31 | 0.344954545 | 0.348219612 | 0.279765662 | 0.28653511 |
| 2002 | 0.6 | 0.372030456 | 0.352584145 | 0.327789647 | 0.291637391 |
| 2002 | 0.43 | 0.393793697 | 0.355259724 | 0.329339169 | 0.285144912 |
| 2002 | 0.42 | 0.400728222 | 0.356065545 | 0.347577597 | 0.271173947 |
| 2002 | 1.45 | 0.39410597 | 0.355748628 | 0.354735037 | 0.259936399 |
| 2002 | 1.49 | 0.379415247 | 0.355148938 | 0.361250922 | 0.264541756 |
| 2003 | 1.5 | 0.362170472 | 0.354907027 | 0.36704484 | 0.286549005 |
| 2003 | 1.45 | 0.348119897 | 0.355780391 | 0.369699911 | 0.310746145 |
| 2003 | 1.48 | 0.342229901 | 0.357383746 | 0.372224566 | 0.323942438 |
| 2003 | 1.18 | 0.344566262 | 0.358083104 | 0.372184377 | 0.329774518 |
| 2003 | 0.89 | 0.34815306 | 0.356933622 | 0.366163095 | 0.338495086 |
| 2003 | 0.68 | 0.344071276 | 0.353817582 | 0.379903359 | 0.351118442 |
| 2003 | 0.96 | 0.329455035 | 0.348994879 | 0.368311158 | 0.359404568 |
| 2003 | 0.88 | 0.310701256 | 0.343314764 | 0.371837263 | 0.357409829 |
| 2003 | 0.01 | 0.296892188 | 0.338153211 | 0.396896227 | 0.347825104 |
| 2003 | 0.83 | 0.289687773 | 0.334270565 | 0.418612677 | 0.338273957 |
| 2003 | 0.52 | 0.282953553 | 0.331248522 | 0.433024167 | 0.334725034 |
| 2003 | 0.33 | 0.27056338 | 0.328470634 | 0.438522394 | 0.338305392 |
| 2004 | 0.43 | 0.255010344 | 0.326420023 | 0.436776968 | 0.345848908 |
| 2004 | 0.48 | 0.244575609 | 0.326253071 | 0.431084495 | 0.351611638 |
| 2004 | 0.61 | 0.24018779 | 0.328403967 | 0.423776108 | 0.349226299 |
| 2004 | 0.57 | 0.234774204 | 0.33259317 | 0.415720587 | 0.334664143 |
| 2004 | 0.88 | 0.222368356 | 0.338364329 | 0.40691933 | 0.309954634 |
| 2004 | 0.04 | 0.201649766 | 0.344898756 | 0.396813883 | 0.286967757 |
| 2004 | 0.44 | 0.174895745 | 0.350848324 | 0.384430971 | 0.284943709 |
| 2004 | 0.85 | 0.149955113 | 0.354568254 | 0.368790456 | 0.311236403 |
| 2004 | 0.75 | 0.143975034 | 0.354628954 | 0.349588503 | 0.349003912 |
| 2004 | -0.11 | 0.170791782 | 0.350339011 | 0.327982788 | 0.378350393 |
| 2004 | -0.63 | 0.217219635 | 0.341814419 | 0.307438633 | 0.393567167 |
| 2004 | -0.17 | 0.259224846 | 0.329982688 | 0.293213391 | 0.397241886 |
| 2005 | 0.44 | 0.287867752 | 0.316616726 | 0.288055115 | 0.392961278 |
| 2005 | 0.81 | 0.304927145 | 0.303308534 | 0.29024193 | 0.383609665 |
| 2005 | 1.36 | 0.314049722 | 0.291138387 | 0.297257577 | 0.371993518 |
| 2005 | 1.03 | 0.318281904 | 0.282028006 | 0.306798571 | 0.360954838 |
| 2005 | 1.46 | 0.319812286 | 0.279209803 | 0.316029785 | 0.351112122 |
| 2005 | 1.17 | 0.319496909 | 0.284605088 | 0.322445367 | 0.340877127 |
| 2005 | 0.66 | 0.316508383 | 0.295875422 | 0.325349029 | 0.329868034 |
| 2005 | 0.25 | 0.309376249 | 0.308435725 | 0.325896335 | 0.319460617 |
| 2005 | -0.46 | 0.297019334 | 0.319280601 | 0.32560336 | 0.312180628 |
| 2005 | -1.32 | 0.279417414 | 0.327488762 | 0.324953493 | 0.309776919 |
| 2005 | -1.5 | 0.259274164 | 0.333022019 | 0.323185772 | 0.31018489 |
| 2005 | 0.2 | 0.240110491 | 0.336173147 | 0.318941272 | 0.310757678 |
| 2006 | 1.03 | 0.221133423 | 0.337553319 | 0.311228718 | 0.312735642 |
| 2006 | 0.66 | 0.20074573 | 0.338186794 | 0.300645109 | 0.317400975 |
| 2006 | 0.05 | 0.183002836 | 0.339455568 | 0.290101642 | 0.320571319 |
| 2006 | 0.4 | 0.176400674 | 0.342440458 | 0.282770107 | 0.316290625 |
| 2006 | 0.48 | 0.187521856 | 0.3466949 | 0.278479089 | 0.302033001 |
| 2006 | 1.04 | 0.216278693 | 0.349745999 | 0.274216788 | 0.279164716 |
| 2006 | 0.35 | 0.25731759 | 0.348462923 | 0.267385227 | 0.252179719 |
| 2006 | -0.65 | 0.304237294 | 0.342517482 | 0.257760767 | 0.227771723 |
| 2006 | -0.94 | 0.352084123 | 0.338416835 | 0.247987815 | 0.212224531 |
| 2006 | -0.05 | 0.397465163 | 0.346844159 | 0.242715553 | 0.207688634 |
| 2006 | -0.22 | 0.437937794 | 0.369944437 | 0.244868805 | 0.21102718 |
| 2006 | 0.14 | 0.471619387 | 0.399450784 | 0.252213349 | 0.217007066 |
| 2007 | 0.01 | 0.496512524 | 0.42752442 | 0.260802103 | 0.222253102 |
| 2007 | 0.04 | 0.509577222 | 0.449493802 | 0.267994505 | 0.226166929 |
| 2007 | -0.36 | 0.50705271 | 0.463015246 | 0.27297164 | 0.229748549 |
| 2007 | 0.16 | 0.486058611 | 0.467973188 | 0.277023262 | 0.233642743 |
| 2007 | -0.1 | 0.447511632 | 0.465859017 | 0.28206101 | 0.23681364 |
| 2007 | 0.09 | 0.401970712 | 0.458894273 | 0.288323248 | 0.237330724 |
| 2007 | 0.78 | 0.373998873 | 0.449442845 | 0.29451824 | 0.234331781 |
| 2007 | 0.5 | 0.38890414 | 0.439519693 | 0.298973664 | 0.22954291 |
| 2007 | -0.36 | 0.443477756 | 0.430242062 | 0.300233424 | 0.227024718 |
| 2007 | -1.45 | 0.506459658 | 0.421527508 | 0.29738982 | 0.230873842 |
| 2007 | -1.08 | 0.552525972 | 0.412503887 | 0.290471303 | 0.242516828 |
| 2007 | -0.58 | 0.57660396 | 0.402595183 | 0.280722156 | 0.259744943 |
| 2008 | -1 | 0.582859342 | 0.392206171 | 0.27024212 | 0.277896089 |
| 2008 | -0.77 | 0.574882049 | 0.382379421 | 0.260983752 | 0.292091485 |
| 2008 | -0.71 | 0.553362384 | 0.37421114 | 0.254076137 | 0.300307887 |
| 2008 | -1.52 | 0.518321795 | 0.368683527 | 0.250038963 | 0.306891991 |
| 2008 | -1.37 | 0.474023323 | 0.366757206 | 0.249050425 | 0.320390449 |
| 2008 | -1.34 | 0.431399943 | 0.369421644 | 0.250030235 | 0.344807134 |
| 2008 | -1.67 | 0.404936892 | 0.377376556 | 0.250242838 | 0.376451406 |
| 2008 | -1.7 | 0.40509145 | 0.390367017 | 0.249034097 | 0.407681521 |
| 2008 | -1.55 | 0.430317038 | 0.407033235 | 0.243206339 | 0.431539696 |
| 2008 | -1.76 | 0.469476237 | 0.425486717 | 0.237252524 | 0.445059124 |
| 2008 | -1.25 | 0.511962481 | 0.443576069 | 0.231647974 | 0.45072385 |
| 2008 | -0.87 | 0.55266618 | 0.458329813 | 0.228998493 | 0.455761868 |
| 2009 | -1.4 | 0.589796413 | 0.467036972 | 0.231120544 | 0.466764017 |
| 2009 | -1.55 | 0.621251055 | 0.470997501 | 0.23844107 | 0.481209182 |
| 2009 | -1.59 | 0.643216331 | 0.473002688 | 0.25060694 | 0.489505228 |
| 2009 | -1.65 | 0.650687805 | 0.472534809 | 0.265985279 | 0.485901732 |
| 2009 | -0.88 | 0.639572872 | 0.468610251 | 0.281889714 | 0.471599325 |
| 2009 | -0.31 | 0.611634719 | 0.461750231 | 0.295385113 | 0.451022953 |
| 2009 | -0.53 | 0.580153599 | 0.453291083 | 0.304368871 | 0.429474186 |
| 2009 | 0.09 | 0.56575043 | 0.444510081 | 0.308551412 | 0.413631565 |
| 2009 | 0.52 | 0.577045349 | 0.435837229 | 0.309166066 | 0.411528157 |
| 2009 | 0.27 | 0.599105747 | 0.426675763 | 0.307678424 | 0.427549978 |
| 2009 | -0.4 | 0.608058024 | 0.416137981 | 0.305324103 | 0.456437088 |
| 2009 | 0.08 | 0.592771964 | 0.404064722 | 0.303587123 | 0.487151874 |
| 2010 | 0.83 | 0.564404906 | 0.391664019 | 0.304417901 | 0.51153481 |
| 2010 | 0.82 | 0.545562267 | 0.381314801 | 0.308942189 | 0.526726275 |
| 2010 | 0.44 | 0.543000665 | 0.375194966 | 0.316066806 | 0.532897566 |
| 2010 | 0.78 | 0.543676613 | 0.373612787 | 0.323201952 | 0.530912383 |
| 2010 | 0.62 | 0.534727356 | 0.374650061 | 0.32793202 | 0.521882095 |
| 2010 | -0.22 | 0.510896386 | 0.375132774 | 0.329110185 | 0.508945365 |
| 2010 | -1.05 | 0.474033654 | 0.371826676 | 0.327371865 | 0.497655296 |
| 2010 | -1.27 | 0.435767317 | 0.362603017 | 0.32514566 | 0.491962225 |
| 2010 | -1.61 | 0.414487468 | 0.349144844 | 0.324914758 | 0.490544565 |
| 2010 | -1.06 | 0.417619772 | 0.341537391 | 0.32594314 | 0.487563019 |
| 2010 | -0.82 | 0.432247115 | 0.351834425 | 0.325504332 | 0.478843697 |
| 2010 | -1.21 | 0.443522469 | 0.375033761 | 0.322998819 | 0.468408142 |
| 2011 | -0.92 | 0.446754931 | 0.397426306 | 0.318842069 | 0.46402592 |
| 2011 | -0.83 | 0.44261399 | 0.412413471 | 0.312380234 | 0.467694578 |
| 2011 | -0.69 | 0.432267377 | 0.419067406 | 0.303468176 | 0.475397317 |
| 2011 | -0.42 | 0.416081791 | 0.419085945 | 0.293025079 | 0.481680623 |
| 2011 | -0.37 | 0.394116412 | 0.41595203 | 0.281533901 | 0.481108385 |
| 2011 | -0.69 | 0.368519207 | 0.413984581 | 0.268761728 | 0.468183772 |
| 2011 | -1.86 | 0.346289249 | 0.416221871 | 0.256057217 | 0.439919453 |
| 2011 | -1.74 | 0.337111319 | 0.422871897 | 0.247079504 | 0.401265988 |
| 2011 | -1.79 | 0.345231592 | 0.431834973 | 0.243789935 | 0.36752927 |
|  |  |  |  |  |  |
